# Elevated nocturnal respiratory rates in the mitochondria of CAM plants: current knowledge and unanswered questions

**DOI:** 10.1093/aob/mcad119

**Published:** 2023-08-28

**Authors:** Alistair Leverett, Anne M Borland

**Affiliations:** School of Natural and Environmental Sciences, Newcastle University, Newcastle upon Tyne NE1 7RU, UK; School of Life Sciences, University of Essex, Wivenhoe Park, Colchester CO4 3SQ, UK; Department of Plant Sciences, University of Cambridge, Downing St., Cambridge CB2 3EA, UK; School of Natural and Environmental Sciences, Newcastle University, Newcastle upon Tyne NE1 7RU, UK

## Abstract

Crassulacean acid metabolism (CAM) is a metabolic adaptation that has evolved convergently in 38 plant families to aid survival in water-limited niches. Whilst primarily considered a photosynthetic adaptation, CAM also has substantial consequences for nocturnal respiratory metabolism. Here, we outline the history, current state and future of nocturnal respiration research in CAM plants, with a particular focus on the energetics of nocturnal respiratory oxygen consumption. Throughout the 20th century, research interest in nocturnal respiration occurred alongside initial discoveries of CAM, although the energetic and mechanistic implications of nocturnal oxygen consumption and links to the operation of the CAM cycle were not fully understood. Recent flux balance analysis (FBA) models have provided new insights into the role that mitochondria play in the CAM cycle. Several FBA models have predicted that CAM requires elevated nocturnal respiratory rates, compared to C_3_ species, to power vacuolar malic acid accumulation. We provide physiological data, from the genus *Clusia*, to corroborate these modelling predictions, thereby reinforcing the importance of elevated nocturnal respiratory rates for CAM. Finally, we outline five unanswered questions pertaining to nocturnal respiration which must be addressed if we are to fully understand and utilize CAM plants in a hotter, drier world.

## INTRODUCTION

**Fig. 1.**
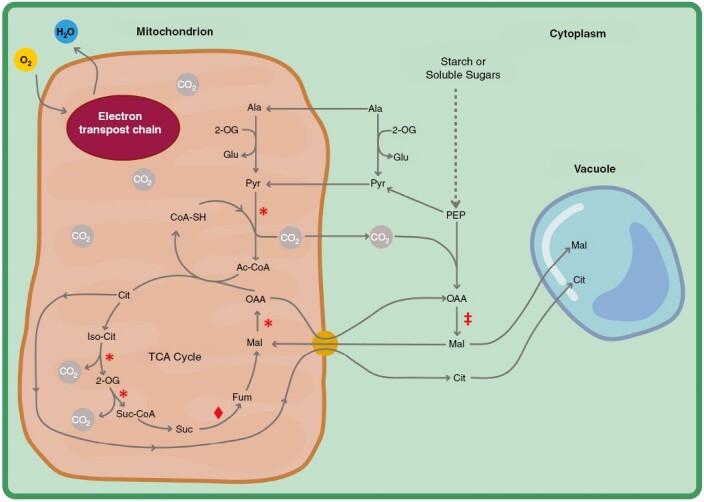
Schematic of major metabolite fluxes involved during the nocturnal phase of CAM and their interaction with the respiratory TCA cycle. Reduction of NAD^+^ to NADH is represented by asterisks; reduction of FAD to FADH_2_ is represented by a diamond; and oxidation of NADH to NAD^+^ is represented by a double dagger. PEP = phosphoenolpyruvate, OAA = oxaloacetic acid, Mal = malate, Cit = citrate, ADP = adenosine triphosphate, ADP = adenosine diphosphate, Pyr = pyruvate, 2-OG = 2-oxoglutarate, Glu = glutamate, Ala = alanine, CoA-SH = coenzyme A, Ac-CoA = acetyl-CoA, Iso-Cit = isocitrate, Suc-CoA = succinyl-CoA, Suc = Succinate, Fum = fumarate. Malate import is depicted to occur via either a malate/OAA import/export protein or via a malate/citrate import/export protein. Organelle sizes are not to scale.

Crassulacean acid metabolism (CAM) is an altered form of photosynthesis that evolved convergently across the vascular plants, primarily as an adaptation to drought (Edwards and Ogburn, 2012; Borland *et al.*, 2018; Heyduk *et al.*, 2019*a*; Gilman *et al*., 2023). CAM is characterized by nocturnal fixation of CO_2_, which occurs predominantly by the action of the enzyme phosphoenolpyruvate carboxylase (PEPC). During the night, PEPC catalyses the carboxylation of the three-carbon molecule, PEP, to the four-carbon molecule, oxaloacetic acid (OAA), which is then converted to malic acid and stored in the vacuole (Fig. 1) (Gilman and Edwards, 2020). In addition, nocturnal citric acid accumulation occurs in several CAM species, although its origin and contribution to diel carbon assimilation remain enigmatic (Lüttge, 1988; Osmond *et al*., 1996; Töpfer *et al*., 2020). During the day, following nocturnal carbon assimilation, malic acid is decarboxylated, by phosphoenolpyruvate carboxykinase, NAD-malic enzyme or NADP-malic enzyme (PPCK, NAD-ME and NADP-ME, respectively) depending on the plant species. The decarboxylation of malic acid regenerates CO_2_ which can then be fixed by Rubisco and enter the Calvin–Benson–Bassham cycle (CBB cycle). Consequently, plants doing CAM are not reliant on diurnal gas exchange to acquire CO_2_ for photosynthesis, which allows their stomata to stay closed during the day (Osmond, 1978; Niechayev *et al*., 2019). In contrast to C_3_ species, which undertake the majority of net CO_2_ assimilation during the day, CAM allows plants to open their stomata predominantly during the night, when vapour pressure deficits are lower, resulting in less water being lost due to transpiration (Haag-Kerwer *et al.*, 1996; Winter *et al*., 2005; Borland *et al.*, 2014). By conserving water during the hottest parts of the day, CAM causes plants to have higher diel water use efficiency (WUE is the moles of carbon gained per mole of water lost) than C_3_ species. Lower diel transpiration rates and elevated WUE together allows CAM plants to survive long- and short-term drought, in drier ecological niches (Bone *et al.*, 2015; Leverett *et al.*, 2021; Schweiger *et al.*, 2021). As a result of the water-saving nature of CAM, there is much interest in bioengineering this pathway into C_3_ crops as a means to prepare for hotter, drier climates of the future (Borland *et al.*, 2015; Sweetlove *et al.*, 2017; Lim *et al.*, 2019; Schiller and Bräutigam, 2021).

CAM is predominantly considered a photosynthetic adaptation, as it begins with the assimilation of CO_2_. However, for this complex metabolic pathway to function efficiently, several auxiliary physio-metabolic adaptations are required. For example, bigger ‘succulent’ photosynthetic cells are needed to store large quantities of malate overnight (Nelson *et al*., 2005; Barrera-Zambrano *et al.*, 2014; Borland *et al.*, 2018; Males, 2018; Leverett *et al.*, 2023*a*), and changes to vascular anatomy are required to meet the altered hydraulic demands of CAM plants (Leverett *et al.*, 2023*b*). In addition, changes to circadian rhythms, stomatal physiology and sugar metabolism are required to support the inverted day/night cycles of gas exchange and provide carbon backbones to feed into the CAM pathway (Dodd *et al.*, 2003; Boxall *et al.*, 2017; Abraham *et al.*, 2020; Ceusters *et al.*, 2021). Adaptations affecting anatomy, stomatal physiology and sugar metabolism have been the focus of several studies and are recognized as integral to the functioning of CAM. However, in recent years less attention has been given to mitochondrial respiration as a co-adaptive trait, within the context of CAM plants. Here, we provide a brief summary of respiration research in CAM plants, focusing on the role of nocturnal oxygen consumption. We then outline recent modelling work that implicates respiratory adaptations as integral to the CAM cycle and provide experimental evidence to corroborate these models. Finally, we argue that respiration research must undergo a renaissance if CAM is to be bioengineered into C_3_ crops in the future.

### The history of respiration research in CAM plants

Studies into respiration go back to the origins of CAM research. In fact, the first study of gas exchange in succulent CAM plants observed nocturnal uptake of O_2_, without the efflux of CO_2_ that is commonly found in C_3_ species (de Saussure, 1804; translated by Hill, 2013). This observation later led Ruhland and Wetzel (1931) to erroneously suggest that nocturnal acidification could be a form of anaerobic respiration, like that seen in animals. However, others refuted this hypothesis. Using excised leaves of various CAM succulents incubated in the dark with different CO_2_ concentrations, Bonner and Bonner (1948) demonstrated a relationship between the concentration of CO_2_ and amount of acid synthesized. From this it was inferred that nocturnal CO_2_ concentrations inside succulent tissues, when acids are accumulating rapidly, must be high as a result of respiratory CO_2_ production inside the leaf (Bonner and Bonner, 1948). Similarly, Thomas (1949) also assumed that CO_2_ concentrations within succulent leaves would be elevated at night because of respiratory CO_2_ production, and that the internal CO_2_ concentration directly determines the rate of acid production. Whilst such studies correctly identified that nocturnal malic acid synthesis is a result of CO_2_ serving as a substrate for the reaction catalysed by PEPC, researchers at this time still considered respiration to be the main source of CO_2_ fixed at night into malic acid. The demonstration of direct uptake of CO_2_ from the atmosphere at night, and the realization that this was mechanistically and stoichiometrically linked to the harvesting of external CO_2_ for photosynthesis, was only made after whole-plant gas-exchange studies (Thomas and Ranson, 1954; Ranson and Thomas, 1960 and references therein). As a result of these early pioneering experiments, CAM was ultimately recognized as a photosynthetic pathway, rather than a respiratory adaptation.

With the discovery that acidification is the result of CO_2_ assimilation in the dark, and that night-time O_2_ uptake is also a phenomenon expected in all aerobic organisms, the mechanistic and energetic links between nocturnal CO_2_ uptake, O_2_ influx and the operation of CAM continued to intrigue researchers particularly since anaerobic conditions prevented nocturnal acidification of plant tissues (Moyse, 1955; Moyse and Jolchine, 1956). Investigations on *Kalanchoë* found that acid leaked out of the vacuole at night under anaerobic conditions, suggesting that respiration is needed to maintain a malate gradient across the tonoplast membrane (Nishida and Hayashi, 1979). This finding was supported by stoichiometric calculations, which found that ATP was needed for the accumulation of malic acid in the vacuole at night (Lüttge *et al.*, 1981). Whilst transport of malate per se across the tonoplast is not ATP-requiring, ATP is needed to drive active transport by the tonoplast H^+^-ATPase with the malate anions then following passively (Smith *et al.*, 1984*a*). Later, the development of technology to isolate intact vacuoles allowed for more direct characterization of the ATPase requirements of tonoplast malate transport, supporting the view that respiration was required to drive this process (Aoki and Nishida, 1984; Smith *et al*., 1984*a*, *b*). Investigating different tissues within a leaf further implicated the importance of high respiration rates to power nocturnal acidification. Lüttge and Ball (1987) compared O_2_ consumption in photosynthetic tissue where CAM occurs to adjacent achlorophyllous water storage hydrenchyma, in which the CAM cycle is absent. Across a number of species, hydrenchyma tissue exhibited lower nocturnal rates of O_2_ consumption than photosynthetic chlorenchyma, suggesting that the presence of CAM is accompanied by elevated respiratory rates (Lüttge and Ball, 1987). Likewise, comparing different species within *Clusia* found that strong CAM was associated with higher nocturnal O_2_ consumption than weak CAM (where only a fraction of total carbon assimilation occurs via the CAM pathway) (Franco *et al*., 1990). However, despite these indications of the importance of nocturnal respiration for CAM, the 21st century has seen a substantial hiatus of experimental research into respiratory rates. This hiatus is, in large part, due to infrared gas analysis (IRGA) becoming a dominant tool for measuring the physiological attributes of CAM. Most IRGA platforms measure CO_2_ but not O_2_ fluxes and since CO_2_ generated from nocturnal respiration can be reassimilated by PEPC, the rate of respiration at night in CAM plants cannot be quantified in this way (Griffiths *et al.*, 1986; Haag-Kerwer *et al.*, 1996; Tcherkez, 2017; Males and Griffiths, 2018). As a result, over the last 20 years, relatively few studies have focused on mechanistic aspects of nocturnal O_2_ consumption and how this interfaces with the operation and energetic requirements of the CAM cycle.

### Current respiration research in CAM plants

Recent advances in metabolic modelling have brought respiratory O_2_ consumption back to the forefront of CAM research. Considerable progress has been made in modelling metabolic flux through the CAM pathway (Cheung *et al.*, 2014; Tcherkez, 2017; Shameer *et al.*, 2018; Töpfer *et al.*, 2020; Tay *et al*., 2021; Burgos *et al*., 2022; Moreno-Villena *et al.*, 2022) with predictive calculations suggesting that the 24-h CAM cycle requires up to 2.2 additional moles of ATP per mole of CO_2_ fixed, compared with C_3_ photosynthesis (Winter and Smith, 1996; Tcherkez, 2017). Stoichiometric predictions of higher ATP demand for CAM are not themselves new and reflect the additional energy costs imposed by: (1) the conversion of pyruvate (or PEP) back to the level of storage carbohydrate during the light phase and (2) vacuolar acidification at night. The additional ATP requirements for nocturnal acidification in most CAM species are believed to be accounted for primarily by the glycolytic breakdown of storage carbohydrate which is synthesized over the previous day (Winter and Smith, 1996). Lüttge *et al.* (1981) demonstrated that for starch-storing CAM plants, some 50 % of the ATP costs of the vacuolar proton pump could be met via substrate-level phosphorylation in glycolysis. The same study suggested that the remaining 50 % of ATP is supplied via oxidative phosphorylation in mitochondrial respiration, which was shown to occur at rates sufficient to supply this ATP (Lüttge *et al*., 1981). In contrast, in CAM species such as *Clusia* in which a substantial part of the daily carbohydrate reserve is free hexose, there is no net nocturnal ATP production in the glycolytic conversion of hexose to PEP, so the entire ATP cost for nocturnal acidification must be met by mitochondrial respiration (Winter and Smith, 1996).

More recently, the CAM pathway has been described using flux balance analysis (FBA) metabolic modelling, which is able to simulate the totality of biochemical reactions in a cell. FBA models have indicated that CAM requires higher flux through the tricarboxylic acid (TCA) cycle and the mitochondrial electron transport chain, in comparison to C_3_ plants (Cheung *et al.*, 2014; Shameer *et al.*, 2018). Specifically, a 1.6-fold higher flux through mitochondrial ATP-synthase is predicted, in part to drive the accumulation of malic acid in the vacuole for overnight storage (Shameer *et al.*, 2018). In addition, FBA has been used to analyse the C_3_–CAM continuum, by modelling intermediate metabolic phenotypes. This approach found that weak CAM employs nocturnal respiratory rates higher than C_3_, but lower than strong CAM. Furthermore, Tay *et al.* (2021) found that flux through both the TCA cycle and mitochondrial electron transport chain increase linearly with greater flux through the CAM cycle. Together, these recent FBA models have reinstated the integral role that elevated nocturnal respiratory rates play in the CAM cycle and have highlighted a central role for mitochondrial metabolism and electron transport in particular. However, despite the substantial history of respiratory research in CAM plants, outlined above, experimental evidence directly comparing nocturnal respiratory rates of oxygen consumption in CAM and C_3_ plants is lacking.

In this study, we sought to provide experimental evidence to corroborate the prediction from FBA models: that CAM requires elevated nocturnal respiratory rates. We opted to study a facultative CAM species which transitions from C_3_ to CAM in response to drought stress, thereby allowing meaningful comparisons to be made between these forms of photosynthesis, within a single species (Winter and Holtum, 2014). We also analysed a closely related, cogeneric obligate C_3_ species in parallel, which does not induce CAM under drought. This allowed us to reveal if any physiological change accompanying the drought-induced shift from C_3_ to CAM is a direct consequence of CAM or simply a more general response to drought (Borland and Griffiths, 1990; Borland *et al.*, 1992; Peckmann *et al*., 2019). The experimental system we used comprised two species from the genus *Clusia*, a genus known for its remarkable photosynthetic diversity (Franco *et al.*, 1990, 1994; Borland *et al.*, 1992; Holtum *et al.*, 2004; Winter *et al.*, 2005, 2009; Barrera-Zambrano *et al.*, 2014; Leverett *et al.*, 2021, 2023*a*; Luján *et al.*, 2021, 2023; Pachon *et al.*, 2022). By comparing nocturnal oxygen consumption in both a facultative CAM and an obligate C_3_ species in *Clusia* we were able to directly test the hypothesis that CAM employs a higher nocturnal respiration rate than C_3_ photosynthesis. Our findings support recent model-based predictions about the role of mitochondrial adaptations required for CAM and thus highlight the need to direct research efforts in deciphering the mechanistic detail of how nocturnal respiratory metabolism is interfaced with operation of this photosynthetic specialization.

## MATERIALS AND METHODS

### Growth conditions

Prior to experimentation, 2-year-old plants were moved from a glasshouse collection in Oak Ridge National Laboratory, Tennessee, USA, to a controlled environment room providing a 12-h day/night cycle at 29/19 °C. Photosynthetic flux density was ~500 µmol m^−2^ s^−1^ at leaf height. Plants remained under these conditions for 2 weeks before experimental treatments commenced. Three individual plants from each species were watered every 3 d (well-watered treatment), whilst water was withheld from a further three individual plants of each species for a period of 22 d (drought treatment). Diel gas exchange was measured between days 19 and 21 of the drought treatment. Oxygen electrode analysis, as well as sampling for metabolite assays, was conducted on the final day of the drought treatment. Final volumetric soil water content values (% v/v) were measured with an SM150 Soil Moisture Kit (delta-T, Cambridge, UK) and are included in Table 1.

**Table 1.** Average volumetric soil water contents (%) during experimental analysis of *Clusia pratensis* and *C. tocuchensis* (*n* = 3).

	Well-watered	Drought-treated
*C. pratensis*	40	4
*C. tocuchensis*	37	3

### *A*/*C*_i_ curves and diel gas exchange

In order to estimate Rubisco carboxylation capacity, A/Ci curves were constructed. Measurements for A/Ci curves were made prior to drought treatment, at a saturating light intensity of 1000 µmol m^−2^ s^1^ (as determined by light response curves). Gas exchange was measured using an LI-6400XT infrared gas analyser (LI-COR, Lincoln, NE, USA: www.licor.com). Photosynthetic assimilation (*A*) was measured for each acclimated leaf under CO_2_ concentrations of 0, 50, 100, 200, 300, 400, 500, 600, 800, 900, 1000 and 1200 ppm and plotted against internal leaf CO_2_ concentrations (*C*_i_) using an Excel *A*/*C*_i_ curve fitting tool (Sharkey, 2016). Following advice from Sharkey (2016), estimates of mesophyll conductance (*g*_m_) were not estimated using curve fitting in this excel tool. Instead, estimates of *g*_m_, based on the isotope fractionation technique from *Clusia minor* and *C. aripoensis* (Gillon *et al.*, 1998), were applied to the *A*/*C*_i_ curve data for *C. pratensis* and *C. tocuchensis*, respectively. For each species, five replicate curves could be fitted well, and thus *n* = 5.

Diel net CO_2_ uptake was measured over a 24-h light/dark period using an LI-6400XT infrared gas analyser, set to track external light and temperature conditions. Data was logged every 10 min. Three individuals of well-watered and three droughted plants were measured for each species.

### Metabolite assays

Leaves were sampled at dawn and dusk. A 30-mm-diameter disc was cut halfway along the leaf lamina, excluding the midrib. Leaf discs were immediately snap frozen in N_2_ before freeze-drying. Tissue was then crushed into a fine powder and heated in 80 % methanol for 1 h. Samples were centrifuged for 15 min at 142 g, the supernatant cleaned with 0.01 g of activated charcoal (BDH Ltd), and re-centrifuged. The supernatant was dried down and resuspended in 200 mmol Bicine buffer at pH 7.8. Malate and citrate concentrations were measured using the biochemical assays of Hohorst and Möllering, respectively (Hohorst, 1970; Möllering, 1985).

### Nocturnal oxygen consumption

Measurements were made during a single night between midnight and 0400 h, using a Hansatech oxygen electrode (Hansatech Ltd, UK: www.hansatech-instruments.com). The oxygen electrode was assembled using 50 % saturated KCl (Sigma) as the electrolyte and held at 19 °C. Inside the chamber one foam disc was kept moist with deionized water and another with 1 m KHCO_3_ (Sigma). From each leaf a 10-cm^2^ disc was cut, avoiding the midrib, and placed in the chamber. Leaf discs were given 5 min to acclimate in the chamber. Once acclimated, O_2_ content was measured every 2.5 s for 5 min. The randomized experimental design, with sampling times, is included as a Supplementary Data file.

###  Statistics

Statistics were performed using R, v.3.6.3 (R Core Team, 2018). Metabolite assay and O_2_ electrode data were standardized both by leaf area and leaf fresh weight.

## RESULTS

**Fig. 2.**
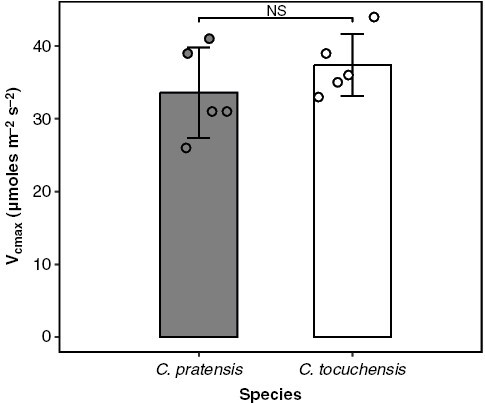
*Clusia pratensis* and *C. tocuchensis* have similar maximal carboxylation capacities (*V*_cmax_). Under well-watered conditions, *V*_cmax_, estimated from *A*/*C*_i_ curves, did not differ between the species studied (two-tailed t-test; *t* = 1.12, *P* = 0.30). For each species, *n* = 5, bars represent mean and error bars represent ±1 standard deviation. Individual replicate measurements are depicted as circles.

The facultative CAM species selected for this study was *C. pratensis*, a species known to exhibit a clear C_3_ or CAM phenotype under well-watered or drought-treated conditions, respectively (Winter *et al*., 2008; Winter and Holtum, 2014; Leverett *et al.*, 2021). We wanted to include an appropriate obligate C_3_ species in our comparison, to ensure that any differences in nocturnal respiratory rates were the consequence of CAM, and not other physiological factors. One potentially confounding variable is Rubisco carboxylation capacity (*V*_cmax_), which is thought to be positively correlated with interspecific differences in nocturnal respiratory rates (Wright *et al.*, 2004; Wang *et al.*, 2020; Iqbal *et al.*, 2021). We constructed *A*/*C*_i_ curves for *C. pratensis* and *C. tocuchensis* (obligate C_3_) (Borland *et al.*, 1992). The inclusion of *C. tocuchensis* in our experiment was justified by the observation that this species has similar photosynthetic physiology to *C. pratensis* under well-watered conditions, as demonstrated by the finding that there was no significant difference in *V*_cmax_ between the two species (Fig. 2). Therefore, any respiratory differences observed between *C. pratensis* and *C. tocuchensis* are unlikely to be the indirect effect of contrasting CBB-cycle physiology, and thus are more likely to be explained by the presence/absence of CAM.

**Fig. 3.**
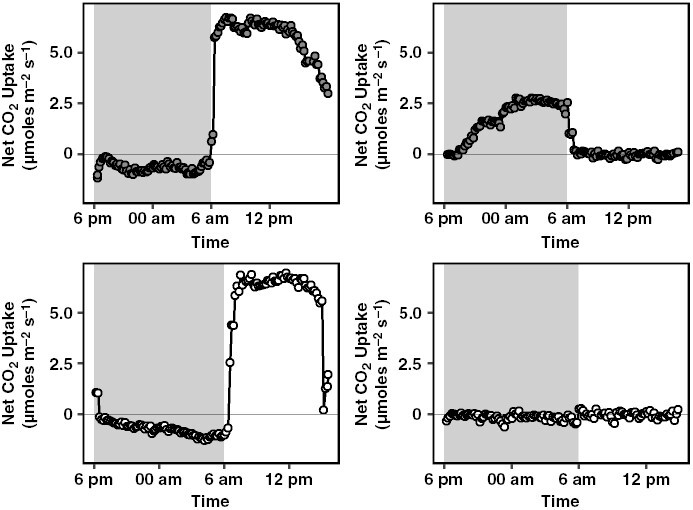
*Clusia pratensis* (grey dots) and *C. tocuchensis* (white dots) exhibit facultative CAM and obligate C_3_ phenotypes, respectively. *Clusia pratensis* diel photosynthetic assimilation rates in (A) well-watered and (B) drought treatments. *Clusia tocuchensis* diel photosynthetic assimilation rates in (C) well-watered and (D) drought treatments. Representative graphs are displayed for gas exchange data.

**Fig. 4.**
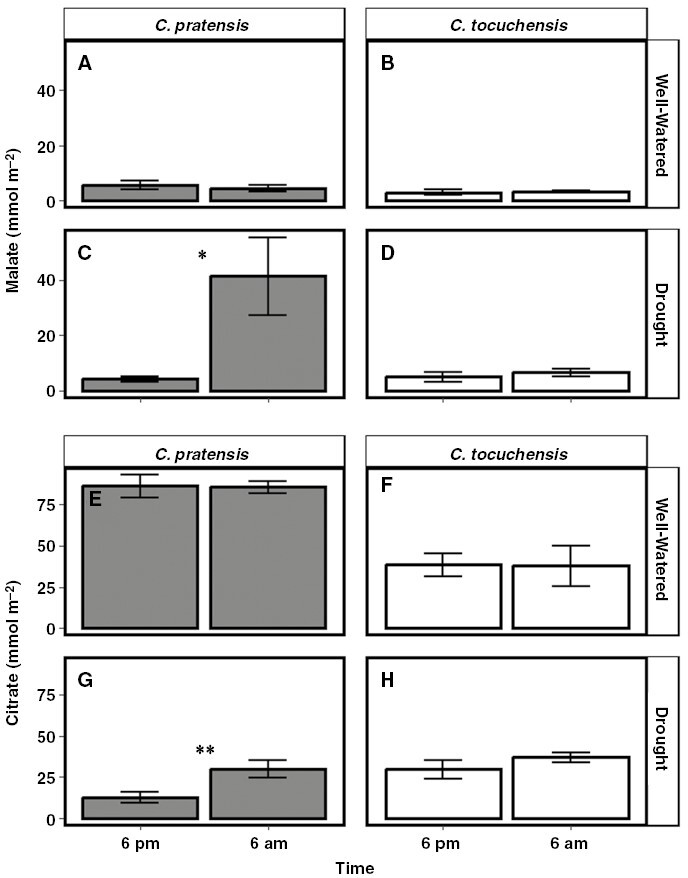
Malate and citrate contents increase nocturnally in drought-treated *Clusia pratensis* plants. (A, B) When plants were well-watered, no significant nocturnal accumulation of malate was detected in *C. pratensis* (*t* = −1.211, *P* = 0.850) or *C. tocuchensis* (*t* = 0.502, *P* = 0.330). (C) When plants were drought treated, a significant nocturnal upregulation of malate was observed in *C. pratensis* (*t* = 4.570, *P* = 0.022) but (D) not in *C. tocuchensis* (*t* = 1.209, *P* = 0.149). (E, F) When plants were well-watered, no significant nocturnal accumulation of citrate was detected in *C. pratensis* (*t* = −0.170, *P* = 0.563) or *C. tocuchensis* (*t* = −0.103, *P* = 0.538). (G) When plants were drought treated, a significant nocturnal upregulation of citrate was observed in *C. pratensis* (*t* = 4.720, *P* = 0.008) but (H) not in *C. tocuchensis* (*t* = 1.976, *P* = 0.071). Error bars represent ±1 standard deviation, and *n* = 3. All *P* values are derived from a one-tailed, independent t-test comparing metabolite content at dawn with that at dusk.

To confirm that *C. pratensis* and *C. tocuchensis* exhibit facultative CAM and obligate C_3_ phenotypes, respectively, net CO_2_ gas exchange was measured over 24 h (Fig. 3). Under well-watered conditions, both species assimilated CO_2_ during the day, characteristic of C_3_ photosynthesis (Fig. 3A, B). Following drought treatment, *C. pratensis* induced a CAM phenotype, characterized by nocturnal CO_2_ assimilation, whereas no such switch occurred in *C. tocuchensis*. Drought also elicited a significant nocturnal accumulation of malate in *C. pratensis* but not in *C. tocuchensis*, confirming that only *C. pratensis* engaged in CAM, when drought stressed (Fig. 4; Supplementary Data, Fig. S1). Substantial reductions in leaf citrate content were observed in CAM-induced *C. pratensis* compared to the well-watered plants (Fig. 4), which is consistent with field observations of citrate mobilisation as CAM is induced in *C. minor* (Borland *et al.*, 1996). Some nocturnal accumulation of citrate was also measured in droughted *C. pratensis* but not in *C. tocuchensis* (Fig. 4).

**Fig. 5.**
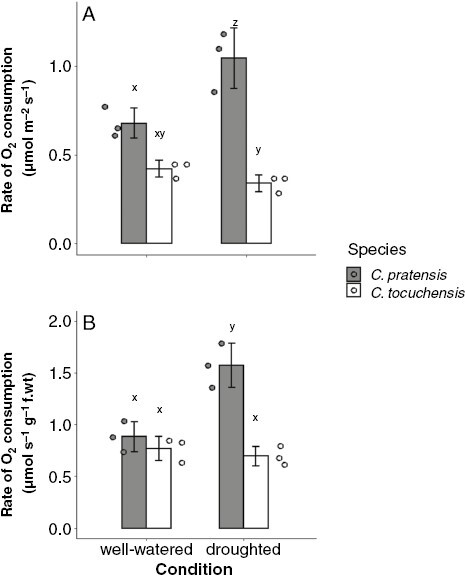
Facultative induction of CAM induces increased nocturnal respiratory rates. Under well-watered conditions, there is a non-significant difference between the O_2_ consumption of either species, such that *C. tocuchensis* is consuming less O_2_. When water was withheld from *C. pratensis* for 22 d, nocturnal O_2_ consumption became significantly higher. When the same drought treatment was applied to *C. tocuchensis*, no significant difference in O_2_ consumption was observed. Significant differences were determined using a two-way ANOVA and post-hoc Tukey–Kramer analysis, with an alpha value of 0.05. Significant groupings are represented by letters above each bar. *P*-values for ANOVA plus each pairwise comparison are available in a Supplementary Table S1. For each species/condition combination, *n* = 3, bars represent mean value and error bars represent ±1 standard deviation. Individual replicate measurements are depicted as circles.

To estimate respiration rates, we measured nocturnal O_2_ consumption using an oxygen electrode and determined significant differences with an ANOVA + Tukey–Kramer analysis (Fig. 5). After 22 d without water, *C. pratensis* exhibited a significant, 1.54-fold increase in nocturnal O_2_ consumption, when compared to well-watered conditions. No significant change in nocturnal O_2_ consumption was observed when *C. tocuchensis* was drought stressed. Therefore, induction of CAM in *C. pratensis* was accompanied by an increase in nocturnal O_2_ consumption, whereas equivalent drought treatment in the obligate C_3_ species, *C. tocuchensis*, caused no such change. The same result was observed when O_2_ uptake was standardized on a leaf area basis and on a fresh weight basis (Fig. 5). Taken together, this two-species comparison within *Clusia* indicates that CAM employs a higher nocturnal respiratory rate than C_3_ photosynthesis.

## DISCUSSION

### Current respiratory research in CAM plants – continued

The experimental system used in this study allowed us to compare rates of nocturnal respiratory O_2_ consumption between CAM and C_3_ in two ways: by comparing C_3_ and CAM physiology within one species (*Clusia pratensis*) and between two closely related species with distinct photosynthetic physiologies (i.e. comparing *C. pratensis* to *C. tocuchensis*). These data show that the facultative induction of CAM in *C. pratensis* was accompanied by elevated nocturnal respiratory rates, which increased by >50 % when plants were drought stressed. No such increase in nocturnal respiration was observed when a comparable level of drought was imposed on the obligate C_3_ species, *C. tocuchensis*. In general, C_3_ plants typically show a very gradual decline in nocturnal respiratory rates from the onset of drought until long after diurnal CO_2_ assimilation has stopped (Slot and Poorter, 2007; Atkin and Macherel, 2009; Crous *et al.*, 2011; Sevanto *et al.*, 2014). Thus, the drought-induced increase in nocturnal respiration in the facultative CAM species, *C. pratensis*, is consistent with the prediction from FBA models that CAM employs elevated nocturnal mitochondrial respiratory rates compared to C_3_ photosynthesis (Shameer *et al.*, 2018; Töpfer *et al.*, 2020; Tay *et al*., 2021).

Our data for nocturnal O_2_ consumption in *C. pratensis* allowed us to calculate if the amount of additional respiratory O_2_ consumption measured in CAM-induced *C. pratensis* was quantitatively sufficient to support the additional energy requirements of nocturnal malate accumulation measured in this species. A net nocturnal accumulation of 37 mmol m^−2^ malate was measured over 12 h (Fig. 4) in CAM-induced *C. pratensis*, and if we assume that sequestration of 1 mol malic acid in the vacuole is energized by 1 mol ATP (Winter and Smith, 1996) this equates to a nocturnal ATP demand of 0.86 µmol ATP m^−2^ s^−1^. The nocturnal respiratory rate of O_2_ consumption in CAM-induced *C. pratensis* increased by 0.37 µmol m^−2^ s^−1^ after drought. This figure for oxygen uptake is multiplied by 2 (to convert to O atoms) and by the P:O ratio, the number of molecules of ATP synthesized per atom of O reduced by an electron pair. Assuming a P:O ratio of 2.5 (see Winter and Smith, 1996), and no change in alternative oxidase activity between treatments, this rate of O_2_ consumption equates to the generation of an additional 1.85 µmol ATP m^−2^ s^−1^ at night after drought which is quantitatively sufficient to support the measured nocturnal accumulation of malate. Assuming that vacuolar sequestration of 1 mol citric acid requires 1.5 mol ATP (Winter and Smith, 1996) our measurements of nocturnal citrate accumulation indicated an additional ATP demand of 0.59 µmol m^−2^ s^−1^. Thus, when *C. pratensis* plants facultatively induce CAM, the increased rate of ATP production calculated from O_2_ consumption (1.85 µmol m^−2^ s^−1^) is sufficient to meet the ATP demand required for the combined accumulation of nocturnal malate and citrate (1.45 µmol m^−2^ s^−1^).

Significant mobilization of citrate was found to accompany the induction of CAM in *C. pratensis*, a phenomenon which was also reported for *C. minor* as CAM was induced during the wet–dry season in Trinidad (Borland *et al.*, 1996). Another *Clusia* species, *C. uvitana*, was also shown to contain high H^+^ contents when operating in C_3_ mode but with CAM induction, an overall decline in acid content was reported (Zotz and Winter, 1994). Could the mobilization of citrate impose additional energetic demands for nocturnal mitochondrial respiration that are independent of the operation of CAM? Diel breakdown/accumulation of citrate is also known to occur in C_3_ plants with citrate required for conversion to 2-oxoglutarate, in order to provide the carbon skeletons needed for light-dependent nitrogen assimilation and amino acid synthesis (Bräutigram *et al.*, 2017). In C_3_ species TCA cycle activity is strongly down-regulated in the light period and conversion of citrate to 2-oxoglutarate occurs in the cytosol through the action of specific isoforms of aconitase and isocitrate dehydrogenase (Hanning and Heldt, 1993; Tcherkez, 2017). In the present study and as reported elsewhere (Borland *et al*., 1996), citrate appears to be mobilized predominantly during the day in *Clusia* (analogous to the situation in C_3_ plants). Thus, it would seem unlikely that drought-induced breakdown of citrate would impose a requirement for elevated nocturnal mitochondrial respiration and that the elevated rates of nocturnal O_2_ uptake measured in droughted *C. pratensis* are indeed a specific requirement for CAM.

### Future respiratory research in CAM plants

Recent FBA models, along with the experimental data described in this study, indicate an important role for elevated nocturnal respiratory rates in the CAM cycle. The future of CAM research is set to be substantially influenced by the desire to bioengineer this metabolic pathway into C_3_ crops as a means of ensuring sustainable growth and productivity in hotter, drier future climates. It is essential that any attempts to bioengineer CAM consider the energetic costs of this metabolic pathway, and the mitochondrial adaptations that are required to facilitate such costs. Looking to the future, we have identified five core questions related to nocturnal mitochondrial respiratory adaptations that must be addressed in order to inform ongoing efforts to bioengineer the CAM pathway into crops.

#### Do all CAM plants show elevated rates of nocturnal respiration?

The data presented in this study focus on two species within the eudicot genus *Clusia*. However, it is important to establish if enhanced flux through the TCA cycle and mitochondrial electron transport chain are common across all CAM taxa. The rapid expansion of genomic datasets for phylogenetically diverse CAM species provides one avenue for examining genes and proteins implicated in nocturnal respiration and mitochondrial metabolism (Tcherkez, 2017). In facultative species where CAM is induced by exposure to salinity and/or drought, comparisons of C_3_ and CAM transcriptomes have shown increased abundance of transcripts for genes implicated in respiratory metabolism, encompassing the reactions of glycolysis, the TCA cycle and mitochondrial electron transport (Cushman *et al*., 2008; Brilhaus *et al.*, 2016). Metabolite profiling can also be informative, as shown in the facultative CAM species *Talinum triangulare* where CAM was elicited by exogenous application of abscisic acid (Malekova *et al*., 2019). They showed that CAM induction was accompanied by nocturnal accumulation of TCA intermediates, suggesting increased flux through the mitochondria. The same study also highlighted a potential role for mitochondrial amino acid metabolism in providing respiratory substrates to satisfy the energetic demands of CAM (Malekova *et al*., 2019). However, as alluded to above, when drought or salinity is used to induce CAM, establishing CAM-specific changes in mitochondrial metabolism from more general stress responses is problematic. Another approach is the comparison of phenotypically diverse genera which encompass C_3_, C_3_–CAM and CAM species. Examples include genera such as *Yucca* (Asparagaceae), *Erycina* and *Dendrobium* (Orchidaceae), which would be ideal models for exploring if elevated mitochondrial respiration is a common feature of CAM in monocots (Heyduk *et al.*, 2019*c*; Li *et al.*, 2019). Likewise, studying the photosynthetically diverse genus *Pyrrosia* (Polypodiaceae) would allow comparisons to be made within the ferns (Chiang *et al.*, 2013; McAdam and Brodribb, 2013).

As genomic resources for CAM expand, it becomes increasingly important to place observations regarding the transcriptome, proteome and/or metabolome in a physiological context. For example, in the genus *Yucca*, both the gene copy number and transcript abundance of mRNA encoding mitochondrial cytochrome *c* (a key component of the electron transport chain) is higher in CAM species than in C_3_ relatives (Heyduk *et al.*, 2019*b*). However, it is unknown if this genetic variation reflects differences in nocturnal oxygen consumption. We propose that measurements of nocturnal O_2_ consumption rates should become an integral component of the physiological screening toolkit used by CAM scientists. Currently, IRGA-based gas exchange systems are used routinely to phenotype different species with contrasting modes of CAM in ecophysiological studies (Winter *et al*., 2008; Holtum *et al*., 2021; Veste and Herppich, 2021), to characterize genetic defects in CAM (Boxall *et al.*, 2017, 2020) and to provide physiological context for projects that focus on comparative ‘-omics’ datasets (Abraham *et al.*, 2016, 2020). By incorporating measurements of nocturnal O_2_ consumption to such ongoing projects, the CAM community can begin to re-establish respiration and mitochondrial metabolism as integral components of CAM physiology.

#### Which components of CAM necessitate elevated nocturnal respiratory rates?

FBA models predict that elevated nocturnal respiratory rates are primarily a consequence of the ATP demand from accumulation of malic acid in the vacuole at night. This prediction could be confirmed, experimentally, by comparing transgenic CAM-defective lines with wild-type (WT) CAM plants such as are available for the model species *Kalanchoë* (Hartwell *et al*., 2016). Such experimental systems would provide unprecedented insight for establishing the specific metabolic components of CAM that require elevated nocturnal respiratory oxygen consumption. For example, knocking out PEPC prevented transgenic plants from fixing CO_2_ into malate at night (Boxall *et al.*, 2020). Therefore, if this knockout line (*KfPPC1-B*) exhibited lower nocturnal O_2_ consumption than the WT, it would provide near-causal evidence that the synthesis of malate, and its downstream import into the vacuole, is linked to elevated respiratory rates. Evidence from PEPC mutants could be reinforced by knocking out the tonoplast aluminium-activated malate transporter (ALMT) responsible for malate transport into the vacuole (Borland *et al.*, 2014), with the hypothesis that elevated respiratory rates are required to power malate sequestration in the vacuole. In contrast, knocking down the decarboxylation enzymes PPCK and NAD-ME via RNAi in *Kalanchoë fedstchenkoi* has been shown to diminish nocturnal malate accumulation, without eliminating it altogether (Dever *et al.*, 2015). Therefore, it is predicted that reduced flux through the CAM cycle in *Kf-rPPDK* and *Kf-rNAD-ME1* lines will impact nocturnal respiratory rates, but the extent of this effect should be less than that in *KfPPC1* (as no malate accumulation occurs in the latter). The generation of transgenic lines with modified starch metabolism could be used to analyse the role that this pathway plays on nocturnal respiration rates (Ceusters *et al.*, 2021). In *Kalanchoë*, starch degradation mediated via the plastidic α-glucan phosphorylase (PHS1) conserves ATP, when compared to the hydrolytic pathway that predominates in C_3_ species (Borland *et al.*, 2016). Consequently, knocking out PHS1 may have little effect on respiratory rates in *Kalanchoë*, if the reduced ATP demand from diminished malate transport is replaced by additional energy requirements of hydrolytic starch degradation. Analysis of the ever-growing collection of *Kalanchoë* transgenic lines would be very informative in establishing mechanistic relationships between CAM and nocturnal respiration rates.

#### What controls the nocturnal partitioning of malate between vacuole and mitochondrion?

Over 40 years ago, C-isotopic labelling studies suggested malate synthesized at night in the cytosol of *Kalanchoë* could enter the mitochondria and exhibit sustained exchange between the vacuole and mitochondrion for several hours after synthesis (Bradbeer *et al.*, 1975; Winter *et al.*, 1982; Kalt *et al.*, 1990). Exchange of isotopic label does not necessarily indicate net flux through metabolic steps and, on the face of it, entry of malate into the TCA cycle at night would appear to compromise net harvesting of CO_2_ into malate in the vacuole. However, these historical findings do pose the questions: (1) What are the main carbon sources that enter the CAM mitochondrion at night to drive respiration? (2) How does the mitochondrion compete for cytosolic carbon at night to fuel respiration whist malic acid synthesis is occurring? In C_3_ species the majority of carbon entering the mitochondria is imported as pyruvate or alanine, with a small minority of import occurring via direct malate transport (Le *et al.*, 2021, 2022). Consequently, experimentally preventing pyruvate and/or alanine import to mitochondria in C_3_ species, so that only malate can enter the mitochondria, results in retarded growth phenotypes (Le *et al*., 2021). It is unclear if this result holds true in CAM plants. Holtum *et al.* (2005) suggested that in CAM species, pyruvate supply to the mitochondria at night might be limited by low activity of pyruvate kinase, which would favour diversion of PEP to PEPC for the synthesis of OAA and thence to malate. However, there is also evidence that pyruvate kinase activity may be sufficient to supply pyruvate to the mitochondria via glycolysis to support the required rates of nocturnal respiration (Winter *et al.*, 1982). Further work is required to examine if and how pyruvate kinase regulates partitioning of PEP between the provision of substrate for CAM or mitochondrial respiration.

The oxidation of malate by NAD-ME and malate dehydrogenase (MDH) could provide pyruvate and OAA required for the decarboxylating steps of the TCA cycle at night (Holtum *et al.*, 2005). Winter and Smith (2022) argued that mitochondrial NAD-ME should be down-regulated during the nocturnal phase of CAM, in order to minimize futile cycling (which would incur extra energy costs) of malate destined for the vacuole. Mitochondrial NAD-ME is known to be critical for daytime decarboxylation of malate in *K. fedtschenkoi* (Dever *et al.*, 2015), but protein abundance of a specific isoform of NAD-ME has been shown to increase at night in this species (Abraham *et al.*, 2020). All malic enzymes show potential for post-translational modification (Schiller and Bräutigam, 2021), which could control their diel activity. Further research is required to establish how NAD-ME is regulated over the day–night CAM cycle and how this equates to measured intercellular fluxes of the products of malate degradation.

Whilst it is theoretically possible that a portion of malate might be decarboxylated at night to supply pyruvate to the mitochondria, the outcomes of FBA models have led to different predictions. Using the FBA model of Shameer *et al.* (2018), with starch or sucrose as the carbohydrate source used to form PEP skeletons for CAM, ~8–10 % of cytosolic malate is estimated to enter the mitochondria (S. Shameer and L. Sweetlove, pers. comm.). Whilst a proportion of malate is diverted from CAM to the mitochondria, this import is offset by export of OAA or citrate, meaning only a tiny fraction (<1 %) of the carbon fixed by CAM is likely to enter the TCA cycle (Shameer *et al*., 2018). Under this scenario, malate import could function to shuttle reducing power from the cytoplasm into the mitochondria, to aid elevated respiratory rates, without making any sizable contributions to the net carbon import. To better determine if malate is supplying carbon, or simply shuttling reducing power, it will be necessary to knock down expression of mitochondrial malate transporters. However, to date, no mitochondrial malate transporters have been unequivocally identified from CAM plants. One interesting candidate gene is Dicarboxylate Carrier 2 (DIC2), which was recently shown to be a malate-import/citrate-export carrier protein in the mitochondria of *Arabidopsis thaliana* (Lee *et al.*, 2021). Steady-state transcript abundance of DIC2 increases with the onset of CAM in the facultative CAM species *Talinum triangulare* and could be important for partitioning cytosolic malate from the cytosol to the mitochondria (Brilhaus *et al.*, 2016). Proteomics analysis has shown that the DIC2 protein shows a clear diel change in abundance in the constitutive CAM species *K. fedtschenkoi* (Abraham *et al.*, 2020). DIC2 protein abundance is highest during the night and lowest during the day in *K. fedtschenkoi* which would be consistent with a proposed role for increasing the import of malate into mitochondria during the nocturnal phase of CAM.

Molecular analysis of the mitochondrial outer membrane would be informative for understanding how this organelle coordinates the transport of metabolites with nocturnal accumulation of malate. The functional identification of mitochondrial membrane carriers remains elusive but the application of techniques such as free flow electrophoresis with LC-MS/MS proteomics look to be promising approaches for characterizing the protein profiles of mitochondrial membranes in CAM species, as demonstrated recently for *Mesembryanthemum crystallinum* (Guo *et al.*, 2021). Alongside such approaches, the diverse transcriptomic and proteomic data sets that have been generated for CAM species in recent years represent a rich and largely untapped resource for shedding light on the presence and abundance of mitochondrial carriers for malate and pyruvate and how these differ between the different modes of CAM, as well as between CAM and C_3_ plants. In addition to considerations of malate import, it is likely that mitochondria in CAM and C_3_ plants have different capacities for malate metabolism. In comparison with C_3_ species, mitochondria from CAM plants exhibit substantial capacity for oxidizing malate to pyruvate (Arron *et al.*, 1979; Spalding *et al.*, 1980) and an increase in capacity of this process was observed following induction of CAM by salinity in *M. crystallinum* (Winter *et al.*, 1986; Peckmann *et al.*, 2012). Further work is required to establish if the mitochondria of CAM and C_3_ plants possess fundamentally different capacities to metabolize malate at night and, if so, to determine what the functional significance of this might be for optimizing the operation of CAM.

#### Can mitochondria fix CO_2_ to form citrate at night?

In addition to considerations regarding respiration, FBA models have recently implicated mitochondrial enzymes as potential contributors to nocturnal carbon fixation in CAM. A model combining FBA and gas exchange predicted that the action of mitochondrial isocitrate dehydrogenase (ICDH) for the nocturnal fixation of CO_2_ to form (iso)citrate was thermodynamically feasible (Töpfer *et al.*, 2020). This prediction comes on the back of a previous hypothesis that nocturnal citrate accumulation in the CAM cycle occurs by ICDH catalysing a reaction in the reverse direction to its typical function (Osmond *et al.*, 1996). Overnight citrate accumulation has long been known to occur in many CAM taxa (including *Clusia*) yet the carbon source of this organic acid has remained enigmatic (Pucher *et al.*, 1947; Lüttge, 1988; Popp *et al.*, 1987; Brilhaus *et al.*, 2016; Pereira *et al.*, 2017). Lüttge (1988) argued against a role for mitochondrial citrate synthesis in net CO_2_ fixation since provision of acetyl-CoA for the citrate synthase reaction via oxidative decarboxylation of pyruvate actually releases CO_2_. This argument, alongside pulse-labelling experiments on *Clusia* (Olivares *et al.*, 1993), indicates that there is currently no experimental evidence to support the hypothesis of ICDH-mediated uptake of CO_2_ to form citrate *in planta* (Winter and Smith, 2022). Citrate accumulation may simply be the result of shuttling reducing power into the mitochondria. If, malate-import/citrate-export carriers are functioning in the mitochondria, citrate accumulation could be a consequence of this membrane transport system, rather than occurring due to ICDH-catalysed CO_2_ fixation. More work is required to understand the role of citrate metabolism in the CAM cycle, and how this links to nocturnal respiration.

#### Does mitochondrial anatomy differ between CAM and C_3_ plants?

A higher flux through the respiratory pathways may require a greater volume and/or number of mitochondria in CAM species. Since Porter *et al.* (1945) generated the first electron microscopy image of a mitochondrion, technologies to visualize organellar morphology and organization have advanced considerably. Consequently, it is now possible to produce 3D renditions of subcellular structures. For example, serial block face scanning electron microscopy (SBF-SEM) works by imaging many sections of a sample, before computationally reconstructing these images into a 3D model (Harwood *et al.*, 2020; Weiner *et al.*, 2021). Another method, cryogenic electron tomography (cryo-ET), does not undertake serial sectioning, but instead tilts a sample and produces 2D images at many different angles, before using these images to reconstruct a 3D model. Both SBF-SEM and cryo-ET are nearly able to achieve isotropic resolutions, which are often <10 nm, which can be used to visualize mitochondrial dimensions (Weiner *et al.*, 2021). In addition, confocal microscopes can be used to generate 3D images of organelles. A method known as plant enzyme-assisted CLARITY (PEA-CLARITY) works by utilizing cell wall degradation enzymes, prior to applying an immuno-localized stain (Palmer *et al.*, 2015). The effect of the enzyme treatment is that both stains and light can penetrate deeper into the sample, ultimately allowing 3D images of cells to be generated without sectioning. The PEA-CLARITY method has recently been used to image C_4_ leaves, to demonstrate that mitochondrial volume and density are higher in bundle sheath cells than the adjacent mesophyll (Fan *et al.*, 2022). These technologies are ideal for investigating mitochondrial morphology and could be used to test several hypotheses in CAM plants. For example, comparing closely related CAM and C_3_ species could establish if CAM is associated with greater mitochondrial volume per cell. In addition, it is possible that the facultative induction of CAM in many species (including *C. pratensis*, in this study) may be accompanied by an increase in mitochondrial volume, to allow additional flux though the TCA cycle and electron transport chain. At an even smaller time scale, it is possible that mitochondrial volume changes over a 24-h cycle. In a constitutive CAM species, *Agave americana*, genes involved in mitochondrial neogenesis and protein import exhibited nocturnal upregulation (Abraham *et al*., 2016). It is therefore possible that mitochondrial volume increases at night to maintain higher respiratory rates required for nocturnal malate transport. The application of these technologies to estimate mitochondrial dimensions and densities would help understand if and how these organelles have adapted to meet the respiratory needs of CAM.

## CONCLUSION

In order to effectively bioengineer CAM, it is essential that we identify all the adaptations that are integral to this photosynthetic specialization. It is unlikely that simply engineering the carboxylation and decarboxylation enzymes (PEPC, PEPCK NAD-ME, etc.) into crop species will suffice, as a number of auxiliary adaptations are required for CAM to function efficiently. As a result, work is currently underway to bioengineer co-adaptive traits required for CAM. For example, leaf succulence has recently been engineered into *Arabidopsis*, which results in larger vacuoles needed for storage of malate (Lim *et al.*, 2018, 2020). In addition to succulent anatomy, it is likely that co-adaptive traits involving mitochondrial metabolism will be needed to meet the energy requirements of CAM. Here, we outline five outstanding questions regarding mitochondrial respiration that should be answered if CAM biodesign is to be achieved. The study of CAM must continue to look beyond photosynthetic pathways towards respiratory functions in order to realize the full potential of this remarkable adaptation to drought.

## SUPPLEMENTARY DATA

Supplementary Information is available online at https://academic.oup.com/aob and consist of the following.

**Figure S1.** Diel organic acid contents, standardized by leaf fresh weight. Including malate content under well-watered conditions and drought treatment, and citrate content under well-watered conditions and drought conditions. Error bars represent ±1 standard deviation. For all measurements, *n* = 3.

**Table S1.** Randomized Experimental Design.

mcad119_suppl_Supplementary_Figure_S1

mcad119_suppl_Supplementary_Table_S1

## DATA AND CODE AVAILABILITY STATEMENT

The data and R scripts that support the findings of this study are available from https://github.com/ali0lev/Clusia_respiration.
