# Supplementary material for: Elevated nocturnal respiratory rates in the mitochondria of CAM plants: current knowledge and unanswered questions
Source: Ann Bot. 2023 Aug 28;132(4):855–67. doi: 10.1093/aob/mcad119 (PMC10799998; doi:10.1093/aob/mcad119)
Supplement: mcad119_suppl_Supplementary_Figure_S1 [file mcad119_suppl_supplementary_figure_s1.docx]

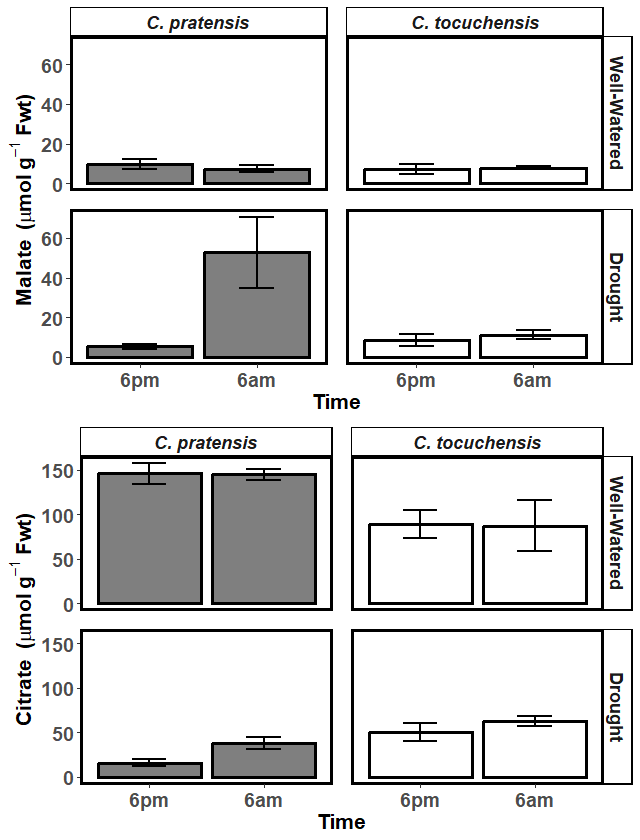


**Figure S1)** Diel organic acid contents, standardised by leaf fresh weight. Including malate content under well-watered conditions and drought treatment, and citrate content under well-watered conditions and drought conditions. Error bars represent ± 1 standard deviation. For all measurements, n = 3.
